# Safety and effectiveness of prilocaine for spinal anesthesia in day surgery setting: a retrospective study on a sample of 3291 patients

**DOI:** 10.1186/s44158-023-00122-6

**Published:** 2023-10-20

**Authors:** Andrea Luigi Ambrosoli, Stefano Di Carlo, Andrea Crespi, Paolo Severgnini, Luisa Luciana Fedele, Vincenza Cofini, Stefano Necozione, Giuseppe Musella

**Affiliations:** 1 Azienda Ospedaliera Di Varese: Aziende Socio Sanitarie Territoriale Dei Sette Laghi, Varese, Italy; 2 ASL 4 Teramo: Azienda Sanitaria Locale 4 Teramo, Teramo, Italy; 3 University of Insubria Faculty of Medicine and Surgery: Università Degli Studi Dell’Insubria, Varese, Italy; 4 University of Aquila: Università Degli Studi Dell’Aquila, L’Aquila, Italy

**Keywords:** Prilocaine, Anesthesia, Spinal, Outpatient surgery, Retrospective study, Perioperative complication

## Abstract

Spinal anesthesia is considered safe and reliable for most surgical procedures involving the lower part of the body, but its use in the ambulatory setting requires drugs with rapid onset and regression of the motor and sensory block-like prilocaine.

The purpose of this study is to retrospectively analyze data from 3291 procedures recorded in our institutional database, to better define the safety profile of spinal prilocaine and the incidence of complications and side effects.

All clinical data, prospectively collected from 2011 to 2019 in an Italian tertiary hospital, of patients treated with spinal anesthesia performed with 40 mg of hyperbaric 2% prilocaine, according to our internal protocol of day surgery, were analyzed.

Surgical procedures included saphenectomy (28.5%, *n* = 937), knee arthroscopy (26.8%, *n* = 882), proctologic surgery (15.16%, *n* = 499), and inguinal canal surgery (14.9%, *n* = 491).

Anesthesia-related complication was represented by urinary retention (1.09%, *n* = 36), lipotimia (0.75%, *n* = 25), and postoperative nausea (0.33%, *n* = 11); arrhythmic events were uncommon (0.18%, *n* = 6). One case of persistent hypotension and 2 cases of persistent hypertension were reported.

Persistent motor or sensory block (lasting more than 5 h) was experienced by 7 patients. One patient (0.03%), who underwent knee arthroscopy, experienced pelvic pain lasting for 6 h, compatible with a transient neurological symptom.

Proctologic surgery was a factor associated with unplanned admission due to anesthesia-related complications (*OR* = 4.9; 95% *CI*: 2–14%).

The number of complications related to the method was low as well as the need for hospitalization. This drug is valid and safe for the most performed day surgery procedures; however, further trials are needed to investigate the incidence of complications in the days following the procedure.

## Introduction

The number of ambulatory surgical procedure is increasing during the last decades, and such trend is expecting to continue in the upcoming years [1]. Spinal anesthesia is considered safe and reliable for most surgical procedure involving the lower part of the body, but its use in the ambulatory and day surgery settings requires drugs with rapid onset and regression of the motor and sensory block, like lidocaine. Due to the high incidence of TNS (transient neurologic symptoms), many institutions abandoned the use of lidocaine, switching to other local anesthetics like low-dose bupivacaine or prilocaine [2]. The latter seems particularly attractive for ambulatory surgery, thanks to a shorter time required for the regression of the block, reducing the risk for unplanned admissions due to persistent block [3].

Several studies confirmed the safety and reliability of spinal prilocaine, but except from one large retrospective study including 5000 patients [4]. The remaining data, including less than 500 patients [5], are derived from small prospective studies that may lack the power to identify side effects with a low incidence.

Since 2011, after the authorization of prilocaine for the Italian market, we developed an internal institutional database for safety surveillance purposes, where data regarding all day-case procedures performed with intrathecal prilocaine were prospectively collected.

In institutional protocol for intrathecal use of hyperbaric 2% prilocaine, a fixed dose of 40 mg was chosen, as suggested by literature [6].

The purpose of this study is to retrospectively analyze data from more than 3000 procedures, to better define the safety profile of spinal prilocaine and the prevalence of complications and side effects.

## Materials and methods

### Study design

We designed a retrospective study to analyze the safety profile and the incidence of side effects associated with prilocaine for spinal anesthesia in ambulatory settings.

All clinical data, collected from 2011 to 2019, of patients treated with spinal anesthesia performed with 40 mg of hyperbaric 2% prilocaine, according to our internal protocol of ambulatory surgery, were analyzed.

The following variables were recorded: age, gender, type of surgery, the presence of surgical, anesthesiological, or general complications, and need for unplanned admission.

When data regarding five patients were missing, additional data were extracted from clinical files stored at our hospital.

### Statistical analysis

Data were summarized with frequencies for categorical data and mean plus/minus standard deviations for continuous data. Chi-square test or Fisher exact test was used to study associations, and confidence intervals were performed. To analyze factors associated with unplanned admission related to anesthesia complication, a logistic regression model was used to compute odds ratio (OR) and 95% CI. All analyses were performed with STATA 14, setting alpha 0.05.

## Results

We analyzed all 3291 patients treated from July 2011 to June 2019, with mean age 50 years (± 24). Varicectomy in the lower extremity/saphenectomy was the more common procedure (*n* = 937), followed by knee arthroscopy (*n* = 882); demographic and surgical data are summarized in Table 1.

**Table 1:** Patient’s characteristics (*n* = 3291)

Variables	*N* or mean	% or SD
Age	49.6	24.2
Male sex	2042	62%
**Surgical procedures**		
Varicectomy/saphenectomy	937	28.4728%
Knee arthroscopy	882	276.8027%
Proctologic surgery	499	15.16%
Inguinal canal surgery	491	14.9115%
Removal of hardware for internal fixation	160	4.8655%
Cysts/lipomas removal	147	4.4655%
Plastic surgery	87	2.6433%
Foot surgery	51	1.5411%
Other orthopedic procedures	28	0.85%
Urologic surgery	9	0.327%

A total of 1.3% (*n* = 44) of our patients required an unplanned admission related to surgical, anesthesia-related, or general complication, as shown in Table 2.

**Table 2:** Reasons for unplanned admission

Reasons for unplanned admission	*n*	%	95% *CI*
Surgical complications	11	0.33	0.2–0.6%
Other complications^a^	33	1%	0.7–1.4%
Anesthesia-related complications	15	0.46	0.3–0.8%
Urinary retention	8	0.24^b^	0.1–0.4%
Postoperative nausea-vomiting	3	0.09^b^	0.03–0.3%
Persistence of the block	5	0.15^b^	0.06–0.4%

^a^Uncontrolled pain, fever, allergic reaction, high blood pressure, hyperglycemia, arrhythmia

^b^% calculated with respect to all admissions

Urinary complications and hemodynamic complications occurred more frequently. Persistent motor or sensory block, defined as a block lasting more than 5 h, was experienced by 7 patients, but only 5 of this were admitted due to persistence of the block for more than 5 h; in 2 cases, a motor block was reported, while in the remaining 5 patients, a sensory block, mainly in the sacral and perineal region, was documented. One patient (0.03%), who underwent knee arthroscopy, experienced pelvic pain lasting for about 6 h, compatible with a transient neurological symptom (Table 3).

**Table 3:** Complications

Complications	*n*	%	95% *CI*
Urinary retention	36	1.09	0.78–1.51%
Hemodynamic complications	34	1.03	0.73–1.44%
Surgical complications	26	0.79	0.05–1.1%
Postoperative nausea-vomiting	11	0.33	0.18–0.60%
Persistence of the block	7	0.21	0.10–0.44%
Headache	2	0.06	0.00–0.24%
Allergic reactions	2	0.06	0.00–0.02%
Fever	2	0.06	0.00–0.02%
Transient neurological symptoms	1	0.03	0.00–0.21%

Among hemodynamic complications which were reported are as follows: 25 cases of lipotomy (0.75%; 95% *CI*: − 0.51–1.12%) and 6 arrhythmic events (0.18%, 95% *CI*: 0.08–0.40%), including 2 cases of sinus tachycardia, 2 atrial fibrillation, 1 atrial flutter and 1 supraventricular tachycardia, 1 case of persistent hypotension, and 2 cases of persistent hypertension (data not in table).

Proctologic surgery was a factor associated with unplanned admission due to anesthesia-related complications (*OR* = 4.9; 95% *CI*: 2–14%); among factors investigated, no other significant associations were found (Table 4). As reference in our center, the overall unplanned admission rate from 2010 to 2019 was 1.21% (1.88% for the patients who received general anesthesia, 1.32% for those who received subarachnoid block with bupivacaine, and 0.17% for those who received peripheral nerve block with or without sedation).

**Table 4:** Factors associated with unplanned admissions related to anesthesia complications

	Unplanned admissions (*n* = 15)	
Covariates	*n* (%)	*p*
Age		
< 18 years	0 (0%)	1.000
18–64 years	13 (87%)	
≥ 65 years	2 (13%)	
Sex		
Female	5 (33%)	0.796
Male	10 (67%)	
Surgical procedures		
Other orthopedic procedures	0 (0%)	1.000
Urologic surgery	0 (0%)	1.000
Plastic surgery	1 (7%)	0.332
Foot surgery	0 (0%)	1.000
Proctologic surgery	7 (47%)	**0.004**
Inguinal canal surgery	2 (13%)	1.000
Cysts/lipomas removal	1 (7%)	0.497
Removal of hardware for internal fixation	1 (7%)	0.527
Knee arthroscopy	2 (13%)	0.381
Varicectomy/saphenectomy	1 (7%)	0.082

## Discussion

Subarachnoid block (SAB) is a validated anesthesiology technique commonly used in clinical practice [7]. Various local anesthetics have been administered over the years in this compartment. The intrathecal use of some of them has been questioned because of complications such as transient neurologic symptoms (TNS), defined as mild to severe pain in the buttocks and legs that appear within a few hours up to 24 h after spinal anesthesia and may last up to 2 to 5 days [8]. Lidocaine in particular was burdened with an incidence of this complication from 10 to 40% [8, 9]. Mepivacaine also had a rather high incidence of this complication (30%) [10]. Other drugs such as bupivacaine and procaine had a rather variable pattern, and the latter, although the incidence of TNS was lower than the other drugs, was still burdened by complications such as nausea, hypotension, or blockade failure [11, 12].

Prilocaine is an amide local anesthetic; its intrathecal use, although it has been tested for several decades, has been introduced relatively recently in Europe. It is hydroxylated to 2-amino-3-hydroxytoluene and 2-amino-5-hydroxytoluene, metabolites responsible for the occurrence of methemoglobinemia. Concentrations of approximately 6 mg/kg are required to determine significant methemoglobinemia [13]. Therefore, dosages of 40–80 mg currently used in SAB in adults should be safe.

The choice to administer a standard dose of 40 mg of prilocaine was dictated by the need to reduce the hemodynamic impact and the incidence of urinary retention which could have led to the patient’s failure to discharge.

Manassero et al. in their literature review identified 40–60 mg of 2% hyperbaric prilocaine for lower limb and lower abdomen surgery for procedures up to 90 min as appropriate [14]. They report anesthetic efficacy and a similar onset time with a significantly lower functional recovery time compared to hyperbaric bupivacaine,16 with a discharge time of 4 h from spinal anesthesia [15]. The faster functional recovery was also associated with a lower incidence of postoperative urinary retention (POUR) [16].

In day-case knee arthroscopy, 40 mg was the dose chosen by Ambrosoli et al. to compare intrathecal blockade with 2% hyperbaric prilocaine versus ultrasound-guided femoral-sciatic nerve blockade with mepivacaine 2%. Sensory and motor blockade recovered sooner after prilocaine spinal anesthesia. Time to home readiness was faster after intrathecal blockade than after peripheral nerve blockade, while time to micturition was not different between the two techniques [17].

In 25 day-case perianal surgeries, Kaban showed one case of POUR after intrathecal 30 mg of 2% hyperbaric prilocaine with fentanyl 20 μg [18].

Some studies report an incidence of complications and the rapidity of recovery after the blockage given by 60 mg of prilocaine superimposable to that given by 40 mg, to the detriment, however, of a lower analgesic extension and a greater need for intraoperative sedation [4, 19].

However, other authors report inconsistent results in terms of the incidence of urinary retention in lower limb minor orthopedic procedures [20]. In our population, where a dose of 40 mg of prilocaine was practiced in all patients, there was no need to resort to intraoperative sedation. Urinary retention was the most frequent complication attributable to anesthetic technique, however in an extremely low percentage (1.09%).

The incidence of other possible complications was also extremely low, confirming the validity of 2% prilocaine for subarachnoid anesthesia in outpatient surgery.

The incidence of such a low number of complications has determined a low need for hospitalization of the patient in the ward; this represents the primary outcome of this analysis which aims to evaluate precisely the low incidence of complications and the patient’s discharge as success of the technique.

The discharge of the patient admitted to the day surgery regime must however take place in compliance with some parameters such as the absence of significant pain and nausea and vomiting; the patient must be able to feed, mobilize actively, and urinate spontaneously. Failure to comply with these parameters therefore leads to the need for hospitalization [21]. In our population, 2% prilocaine at a dose of 40 mg administered in the subarachnoid space allowed the discharge of the majority of patients. Unplanned admission rates reported by the recent literature vary from 0.11 to 2.89% [22, 23] for most day surgery procedures but is higher (up to 6%) for proctologic surgery [24]: the unanticipated admission rates reported by different studies should be compared with caution, since demographical, surgical, and organizational factors can greatly influence such rates.

The association of proctologic surgery with anesthesia-related unplanned admission can be explained by two considerations: the first is that since urinary retention was the more common complication, some cases of urinary retention were probably due to proctologic surgery itself rather than to spinal anesthesia; the second is that the relatively higher dose of local anesthetic delivered to the sacral nerves during the “saddle block” can account for the delayed regression of the block.

### Limitations

Our study does have several limitations: being derived from a retrospective single-center study, our results cannot be automatically generalized to other centers or to other populations, since unplanned admission rates are highly influenced by the patient’s age and the type of surgical procedure.

Retrospective analysis did not allow to analyze some patient characteristics, such as anthropometric values. We believe that they reflect the characteristics of the general population since the sample of patients analyzed is large. Our study does not analyze the incidence of long-term complications, the presence of pain at home, and the need for readmission for complications after discharge. This deficiency is related to the characteristics of our database, which was anesthesia oriented. However, the outcome of our study is not to evaluate the incidence of distant complications or the presence of persistent symptoms after surgery but to verify whether spinal anesthesia with 2% prilocaine is a valid and safe technique for day surgery.

## Conclusion

Given the number of ever-increasing surgical procedures that are performed in day surgery, the use of anesthetic drugs that allow a rapid functional recovery is essential in order to be able to discharge the patient safely and reduce the incidence of complications and consequently the need for hospitalization and related costs. Subarachnoid blockade is a safe method, although there are conflicting opinions on the best type of anesthetic and dosage. In this paper, we report our experience on the use in day surgery of hyperbaric prilocaine 2%. In our experience, the drug has demonstrated a valid anesthetic efficacy with 40 mg, and the number of complications related to the method was low as well as the need for hospitalization. We believe that this drug is valid and safe for the most performed outpatient and day surgery procedures; however, randomized clinical trials are needed which confirm our results and which exclude the incidence of complications in the days following the procedure.

## Data Availability

The datasets analyzed during the current study are available from the corresponding author on reasonable request.
